# Envisioning AI-empowered ecological, environmental, and health protection: Welcome submissions of interdisciplinary research

**DOI:** 10.1016/j.eehl.2026.100266

**Published:** 2026-07-21

**Authors:** Jingwen Chen, Haobo Wang, Wenjia Liu, Jiale He, Guijia Bi, Jiaxing Xie

**Affiliations:** School of Environmental Science and Technology, Dalian University of Technology, Dalian, 116024, China

Eco-Environmental systems are dynamic and interconnected, while health outcomes often arise from multi-scale interactions among exposure and biological response. The increasing uncertainty of climate change, the expansion of chemical use, together with the growing burden of long-term, low-dose and mixture exposures are challenging conventional research paradigms. AI offers new opportunities to integrate environmental monitoring, exposure assessment, chemical analysis, toxicological evidence and cohort data, thereby supporting early prediction, deep mechanistic understanding and proactive risk prevention.

Recent advances in deep learning, generative AI, large language models and AI agents are shifting environmental and health research from isolated analysis toward integrated prediction, reasoning and decision support. AI technologies can link remote sensing images, monitoring networks, chemical structures, spectral data, toxicological assays, omics profiles, epidemiological data and scientific literature into more comprehensive knowledge systems. More importantly, they may enable environmental and health protection to move beyond retrospective assessment toward early warning, source prevention, intelligent control and adaptive governance.

In this context, the journal *Eco-Environment & Health* (EEH) is committed to bridging AI, ecology, environmental science and technology, chemistry, toxicology and public health. By promoting the integration of environmental research with advanced AI technologies, EEH aims to foster new concepts, new models, new methods and new tools for understanding, predicting and controlling environmental and health risks. AI can reshape environmental and health research in several ways, as shown in Fig. 1.

**Fig. 1:**
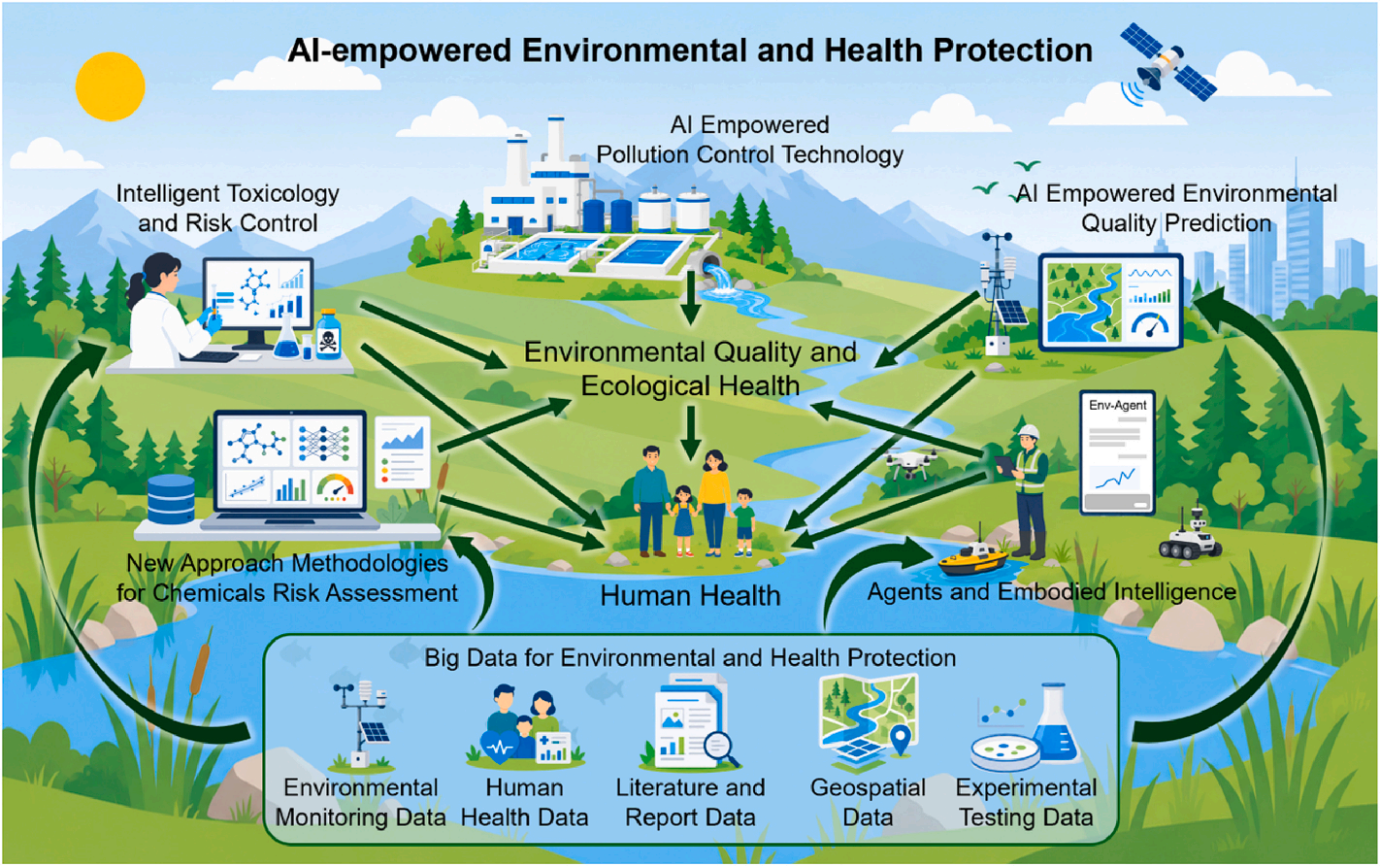
Application of AI in environmental and health research.

## 1 AI-empowered ecological and environmental quality prediction

Ecological and environmental quality prediction provides a foundation for risk identification, early warning, ecosystem conservation and human health protection. With the development of space-air-ground integrated monitoring systems, satellite remote sensing, unmanned platforms, ground sensors, and ecological observation networks are generating high-resolution data on atmospheric conditions, land surface dynamics, water quality, habitat change, biodiversity patterns, ecosystem health, and human activities. AI can transform these heterogeneous data into dynamic predictions of environmental change, ecological degradation, and climate-related risks. One representative trend is AI-based forecasting of large-scale environmental processes. For example, the Pangu-Weather demonstrated that deep neural networks trained on long-term global reanalysis data can rapidly predict medium-range weather and extreme events, suggesting new opportunities for climate-risk assessment and environmental emergency response [1].

In parallel, chemical monitoring is also being transformed by AI-assisted mass spectrometry [2]. Models such as DeepMet [3] and MSGo [4] show that chemical language models and structure generators can help interpret unknown spectra, prioritize candidate structures and accelerate discovery of metabolites and emerging contaminants. Together, these advances suggest that AI-empowered prediction could connect macro-scale environmental and ecological dynamics with micro-scale chemical exposure signatures, thereby supporting comprehensive environmental surveillance, ecological risk assessment, and health risk early warning.

## 2 New approach methodologies for chemicals risk assessment

Understanding the links between chemical exposures and adverse outcomes in ecosystems and humans remains a central challenge in environmental and health research. The growing number of chemicals far exceeds the capacity of traditional testing approaches. In response, new approach methodologies (NAMs) are reshaping chemical risk assessment from a reliance on single-endpoint testing toward an integrated framework that combines mechanistic understanding, experiments and computational prediction [5]. Encompassing *in silico*, *in chemico* and *in vitro* approaches, NAMs are becoming instrumental in constructing an evidence-evaluation framework tailored to both ecological and human relevance [6]. The convergence of microphysiological systems with computational modeling can drive the development of digital twins and virtual tissues, which can dynamically simulate complex systemic responses to bridge molecular-level perturbations and organ-level toxicity.

Such frameworks can support efficient assessment of ecological and health risks posed by chemicals, while also providing methodological support for extrapolating toxic effects across species, exposure scenarios and biological levels of organization. NAMs have also been incorporated by international organizations and regulatory agencies as an important technical route for next-generation chemical risk assessment and regulatory science [5,6]. Intelligent toxicology can further translate model-derived signals into testable hypotheses and decision-relevant evidence, thereby advancing chemical risk assessment towards a more efficient and reliable paradigm.

## 3 Intelligent toxicology and risk control

AI is rapidly transforming toxicology from a largely descriptive discipline into a predictive, mechanistic and design-oriented science. By integrating chemical structures, high-throughput screening data, omics profiles, bioimaging data and toxicological knowledge, intelligent toxicology can enable identification of relationships between chemical exposure and adverse outcomes across molecular, cellular, tissue, organ, population, and ecosystem levels.

Recent advances in multimodal learning, foundation models and self-supervised learning are expanding the scope and performance of conventional computational toxicology [7,8]. These approaches can connect chemical structure with biological response, improve hazard prediction for data-poor chemicals and reveal latent mechanisms that are difficult to capture through conventional methods alone. In the meantime, AI can help prioritize chemicals for testing, identify susceptible populations and support interpretation of complex mixture effects, thereby strengthening the scientific basis of ecological and health risk assessment.

Importantly, AI also creates opportunities to move risk management upstream. Beyond predicting hazards, generative AI can be used to design chemicals and materials that retain desired functionality while reducing persistence, bioaccumulation or toxicity [9]. By integrating hazard prediction and molecular design within a unified framework, intelligent toxicology has the potential to support safe chemical innovation, reduce regrettable substitution and promote source-oriented prevention of environmental pollution [10].

## 4 AI-empowered pollution control technology

Although source prevention is essential, it cannot fully eliminate legacy contamination or unavoidable emissions. Therefore, AI-empowered ecological and environmental protection should advance pollution control and remediation technologies by capturing the nonlinear interactions among contaminants, materials, microorganisms, hydrodynamics, operating conditions, energy use, and by-product formation [11,12]. Beyond removal efficiency, future pollution control should also consider energy consumption, chemical inputs, carbon emissions, resource recovery, secondary pollutants, and ecological effects. By integrating digital twins and adaptive control systems, AI can shift pollution control from empirical optimization toward predictive, low-carbon and intelligent intervention.

## 5 Agents and embodied intelligence for ecological, environmental, and health protection

The rise of large language models has expanded the role of AI from task-specific prediction to reasoning, planning and orchestration [13]. Scientific agents can interpret literature, databases, code, models and multimodal evidence, thereby supporting systematic evidence synthesis, chemical prioritization, NAM-based testing strategies, environmental monitoring and pollution-control decision-making [14]. Multi-agent systems may coordinate specialized models and tools for exposure assessment, toxicological inference, engineering optimization and health impact evaluation.

Embodied intelligence further extends these capabilities from digital reasoning to physical interaction with the environment. When connected to sensor networks, drones, autonomous laboratories, robotic sampling systems and treatment facilities, AI agents may form closed-loop systems that detect problems, design solutions, execute experiments and optimize interventions through continuous feedback [15]. Such systems could transform ecological, environmental, and health protection from human-driven workflows into more automated, adaptive and intelligent operations.

## 6 Big data for ecological, environmental, and health protection

All the AI advances depend on high-quality data. Ecological, environmental, and health research has entered an unprecedented era characterized by a “data deluge”, with rapidly growing information from remote sensing, ecological monitoring, environmental surveillance, omics technologies, epidemiological cohorts, and electronic health records. However, data volume alone does not guarantee scientific value. Current data remain fragmented, heterogeneous and uneven in quality. Standardization, metadata annotation, quality control, data security, interoperability and FAIR (findability, accessibility, interoperability, and reusability) data practices are essential for trustworthy AI.

Big data should therefore be regarded not only as a resource, but also as scientific infrastructure. Knowledge graphs, benchmark datasets, open databases, literature-mining systems and multimodal data platforms will be critical for reproducible model development and cross-disciplinary collaboration. Without such foundations, AI models may remain powerful in appearance but fragile in application.

## 7 Future directions

AI will have a profound impact on global economic and social development as well as the progress of human civilization. The year 2026 is widely recognized as the “Year of Large Model Deployment”, and can also be called the “Year of AI Agents”. AI-powered transformation across industries has only just begun, with boundless room for research and exploration. AI4S (AI for Science) is regarded as the fifth paradigm of scientific research, and has fully transitioned from proof of concept to practical deployment. It’s not just AI4S — AI for engineering, education, and everything else is also evolving and advancing rapidly. Promoting interdisciplinary research on the cutting edges of AI, ecology, environmental science and technology, and health protection, and publishing high-level innovative studies in related directions, has thus become one of the core missions of EEH.

Through this Editorial, EEH invites interdisciplinary studies on AI-empowered environmental quality prediction; new approach methodologies for chemical risk assessment; intelligent toxicology and risk control; AI-empowered pollution control technology; agents and embodied intelligence for ecological conservation, environmental and health protection; and big data for environmental and health protection.

Novelty of research methodology, quality and size of datasets, scientific rigor and robustness of modeling procedures and models, and magnitude of impact on eco-environmental and health protection are core factors in determining whether a submitted manuscript will be accepted for publication. Through these endeavors, EEH will foster a platform for AI-driven interdisciplinary research to advance environmental-health protection and contribute to sustainable development.
